# Remote Activation of the Wnt/β-Catenin Signalling Pathway Using Functionalised Magnetic Particles

**DOI:** 10.1371/journal.pone.0121761

**Published:** 2015-03-17

**Authors:** Michael Rotherham, Alicia J. El Haj

**Affiliations:** Institute for Science and Technology in Medicine, Keele University, Stoke-on-Trent, United Kingdom; University of Texas Medical Branch, UNITED STATES

## Abstract

Wnt signalling pathways play crucial roles in developmental biology, stem cell fate and tissue patterning and have become an attractive therapeutic target in the fields of tissue engineering and regenerative medicine. Wnt signalling has also been shown to play a role in human Mesenchymal Stem Cell (hMSC) fate, which have shown potential as a cell therapy in bone and cartilage tissue engineering. Previous work has shown that biocompatible magnetic nanoparticles (MNP) can be used to stimulate specific mechanosensitive membrane receptors and ion channels in vitro and in vivo. Using this strategy, we determined the effects of mechano-stimulation of the Wnt Frizzled receptor on Wnt pathway activation in hMSC. Frizzled receptors were tagged using anti-Frizzled functionalised MNP (Fz-MNP). A commercially available oscillating magnetic bioreactor (MICA Biosystems) was used to mechanically stimulate Frizzled receptors remotely. Our results demonstrate that Fz-MNP can activate Wnt/β-catenin signalling at key checkpoints in the signalling pathway. Immunocytochemistry indicated nuclear localisation of the Wnt intracellular messenger β-catenin after treatment with Fz-MNP. A Wnt signalling TCF/LEF responsive luciferase reporter transfected into hMSC was used to assess terminal signal activation at the nucleus. We observed an increase in reporter activity after treatment with Fz-MNP and this effect was enhanced after mechano-stimulation using the magnetic array. Western blot analysis was used to probe the mechanism of signalling activation and indicated that Fz-MNP signal through an LRP independent mechanism. Finally, the gene expression profiles of stress response genes were found to be similar when cells were treated with recombinant Wnt-3A or Fz-MNP. This study provides proof of principle that Wnt signalling and Frizzled receptors are mechanosensitive and can be remotely activated in vitro. Using magnetic nanoparticle technology it may be possible to modulate Wnt signalling pathways and thus control stem cell fate for therapeutic purposes.

## Introduction

Wnt signalling is a complex pathway involved in the regulation of a range of biological processes ranging from cell proliferation and differentiation to embryogenesis, tissue formation and regulation of stem cell niches [1] [2]. In humans Wnt proteins consist of a class of nineteen evolutionary conserved, glycosylated and lipid modified proteins each harbouring a cysteine rich domain [3] [4]. The primary receptor for Wnt ligands are the Frizzleds, a family of ten G-protein like 7- transmembrane spanning receptors [5]. Frizzleds have an extended N-terminal region harbouring a cysteine rich domain (CRD) which is required for Wnt reception [3] [4] [6]. In Canonical Wnt/β-catenin signalling, Wnt proteins interact with a receptor complex comprised of Frizzled (Fz)/Low density lipoprotein (LDL) receptor-related protein (LRP) located on the cell membrane [7] [8] [9]. When activated by Wnt ligands, LRP becomes phosphorylated at multiple sites. The activated Fz/LRP receptor complex recruits Axin to the cell membrane [10] [11]; this causes the sequestration of several other intracellular proteins required for Wnt signalling modulation, including glycogen synthase kinase-3β (GSK-3), Dishevelled (Dsh) and Adenomatous Polyposis Coli (APC). In the absence of an external Wnt signal GSK-3, Dsh and APC form a destruction complex that regulates the cytoplasmic pool of the transcriptional regulator β-catenin [4] by successive phosphorylation which marks β-catenin for proteasome degradation [12] [13]. In the presence of a Wnt signal the destruction complex dissociates and is deactivated; as a result active β-catenin accumulates in the cytoplasm and nucleus. Nuclear β-catenin acts as a transcriptional regulator and interacts with the lymphoid enhancer-binding factor 1/T-cell specific transcription factor (LEF/TCF) family of transcription factors which bind to and activate Wnt responsive genes with TCF/LEF binding sites [4].

Human Mesenchymal Stem Cells (hMSC) are multipotent stem cells involved in bone and cartilage formation during development. As such, these cells are of great interest for orthopaedic tissue engineering [14] [15]. Wnt signalling has been shown to elicit different outcomes on cell fate depending on the cell type and Wnt concentration [16] [17]. Human MSC have been shown to express a number of Wnt ligands including Wnt 2, 4, 5a, 11 and Wnt 16 along with Frizzled receptors 2–6 and Wnt inhibitors sFRP 2–4 and Dkk1 [18]. Activation of canonical Wnt signalling by Lithium or Wnt3A has been shown to inhibit MSC differentiation whilst promoting proliferation and maintaining multi-potency [19] [20] [21]. However in certain contexts canonical Wnt signalling has been shown to promote osteogenesis. For example, Wnt3A has been shown to promote osteogenesis in calvarial osteoblasts [16] and hMSC and overexpression of LRP6 / stabilised β-catenin has been shown to enhance osteogenesis In hMSC [17] [22] [23]. The Wnt pathway has also been shown to be mechanoresponsive. Mechanical stimulation of hMSC using oscillatory fluid flow has been shown to promote osteogenesis and cause up-regulation of Wnt5a and the Wnt co-receptor Ror2 as well as promoting β-catenin mobilisation [24].

Magnetic nanoparticles (MNP) have many applications in various fields of science, technology and medicine. For a detailed review of the applications of MNP in biomedicine see Pankhurst *et al* [25]. MNP can be functionalised with biomolecules such as peptides and antibodies and used to target, stimulate and activate specific mechano-sensitive cell receptors and signalling pathways. Remote activation is achieved by applying an oscillating external magnetic field to cause a translational torque in the MNP which is then transduced to the target mechanosensitive protein [26] [27] [28].

MSC have been shown to be load responsive *in vitro* and *in vivo*. Previous work using bio-functionalised MNP targeted to the mechano-responsive ion channel TREK 1 and integrin receptors (using RGD) has shown that it is possible to promote an osteochondral phenotype in hMSC *in vitro* and *in vivo* [29] [30]. This raises the question of what other cell surface receptors are Mechanosensitive and what the effects on cell signalling pathways are when these receptors are stimulated. In the context of Wnt signalling, one of the drawbacks of using recombinant Wnt protein is the limited availability of large quantities of bio-active growth factor. This is partly due to the biochemical nature of Wnt’s, which have extensive post-translational modifications that are required for their bio-activity. These modifications make Wnt proteins hydrophobic in nature and therefore affect their stability in solution [31] [32]. One way of circumventing these problems is to artificially target and activate Wnt receptors directly using MNP technology. To date no one has studied the mechano-sensitivity of Wnt receptors and the effect of mechano-stimulation on Wnt signalling activity in hMSC.

## Materials and Methods

### Cell culture

Fresh human bone marrow (Lonza, cat. no. 1M-125) was sourced from healthy volunteers with written, informed consent obtained by Lonza. No ethical approval was required for this project as the human tissue (bone marrow aspirate) was acquired from a commercially available source. The donor program is approved annually by a commercial institutional review board and Lonza Walkersville Inc. is a licensed tissue bank. The adherent cell population was selected from fresh bone marrow by culturing aliquots of aspirate for 2 weeks in Low glucose DMEM with 5% FBS, 1% L-glutamine and 1% Penicillin and Streptomycin with media changes performed once per week. MSC were routinely characterised for expression of surface markers (CD73+, CD90+, CD105+ and CD45-, CD34-, CD14-, CD19- and HLA-DR-) and histological staining for osteogenic (Alizarin red), chondrogenic (Alcian blue) and adipogenic differentiation (Oil red O) (Data not shown). For cell expansion MSC were cultured in high glucose DMEM supplemented with 10% FBS, 1% L-glutamine and 1% Penicillin / Streptomycin (All reagents Lonza). Cells were passaged once per week and cells between passage 2 and 5 were used in all experiments.

### Transient transfections

For Wnt luciferase reporter studies, hMSC were transiently transfected with a Gaussia luciferase reporter gene under control of an 8x TCF/LEF promoter (provided by Dr Hu Bin and Neil Farrow, Keele University). Cells were seeded into 24-well plates and transfected with 0.5μg/well of TCF/LEF reporter plasmid. Transfections were performed in reduced serum opti-MEM media without antibiotics using 2.25μL Lipofectamine LTX with 0.5μL Plus reagent per well (All Invitrogen). After 4h media was aspirated and replaced with fresh antibiotic free media.

### MNP coating

250nm SPIO carboxyl functionalised magnetic nanoparticles (Micromod) were covalently coated with anti-Frizzled 2 (Abcam), Trek1 (Alomone labs), Rabbit-IgG antibodies (Abcam) or RGD tri-peptide (Sigma) by carbodiimide activation as described previously [33]. Briefly, particles were activated using EDAC and NHS dissolved in 0.5M MES buffer pH6.3 (Sigma) for 60 mins. at room temperature with constant mixing. The particle suspension was washed and re-suspended in 0.1M MES buffer containing 40μg of anti-rabbit secondary antibody (Abcam) or 50μg RGD. The particle suspension was continuously mixed overnight at 4°C then washed and re-suspended in 0.1mL MES buffer containing 10μg of either anti-Frizzled 2 antibody (Abcam), anti-Rabbit-IgG (Abcam) or Anti-Trek1 antibody (Alomone labs). Particle suspensions were mixed for a further 3h at room temperature then blocked with 25mM Glycine (Sigma) for 30mins before final washing and re-suspension in 0.1% BSA in PBS (Fisher). Functionalised nanoparticles were then analysed for surface charge and size using a Zetasizer 3000 HSa (Malvern Instruments). Particles were diluted in dH_2_0 and measurements performed at 25°C. The size and surface charge of coated nanoparticles was compared to uncoated activated nanoparticles.

### Cell labelling with MNP

Media was aspirated and cells washed with PBS. All cells were cultured in basal serum-free MSC media for 2–3h. Particles were added to appropriate groups at approximately 3μg MNP/cm^2^ of culture surface area, and incubated for a further 1.5–3h with intermittent agitation. Media was aspirated and cells washed with PBS to remove unbound particles before addition of fresh media (Serum free for western blotting experiments, 2.5% FBS for luciferase experiments, 10% FBS for gene expression experiments). For positive control groups either recombinant human Wnt 3A (20ng/mL) or diluted Wnt 3A conditioned media (50% or 20%) collected from Wnt 3A overexpressing L-M(TK-) cells (LGC standards) was used.

### Magnetic stimulation

**Fig 1:**
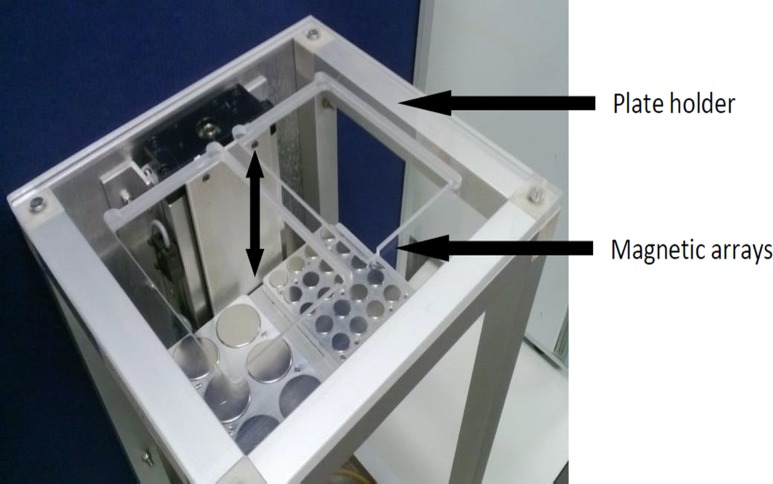
Magnetic Force Bioreactor. Image of the Magnetic Force Bioreactor used in magnetic stimulation experiments. Culture plates are situated on the plate holder above the magnetic array which oscillates vertically beneath the culture plates. The movement parameters for the array are computer controlled.

Magnetically stimulated groups were placed in a commercially available vertical oscillating magnetic force bioreactor (MICA Biosystems) (Fig. 1) situated inside an incubator maintained at 37°C, 5% CO_2_. Non-stimulated control groups were kept in identical conditions (without magnetic field). Magnetically stimulated groups were exposed to a magnetic field of 25–120mT from an array of permanent magnets (NdFeB) situated beneath the culture plates at a frequency of 0.9–1Hz. Magnetic field treatment was applied in 1hr and 3h sessions.

### Western blotting

Cells were lysed with RIPA buffer containing a protease and phosphatase inhibitor mix (Sigma). Cell lysate was clarified and the total protein of each sample was quantified using a BCA assay kit (Fisher). For PAGE, 30μg of protein was mixed with LDS sample buffer with added β-mercaptoethanol (Invitrogen). Samples were then heated for 10mins at 70°C and briefly centrifuged before being loaded onto a Tris-Glycine 4–20% gel in Tris-Gly running buffer (NuSep). Proteins were transferred to PVDF membrane (Fisher) which was then blocked with 5% milk powder (Co-operative) in TBST buffer (Sigma). The membrane was then incubated with anti-LRP6, anti (Ser1490) phospho-LRP6, anti-LRP5 (all New England Biolabs), anti-phospho-LRP5 (Abnova) or anti-GAPDH (Abcam) overnight at 4°C with constant mixing. The membrane was washed with TBST 3x before incubation with Anti-rabbit-HRP or anti-Mouse-HRP (1:1000) (Abcam) for 1h at room temp. The membrane was washed 5x in TBST and chemiluminescence developed using a PicoWest chemiluminescent kit (Thermo Pierce) followed by image capture using a Protein Simple Flourchem M imaging system.

### Immunocytochemistry

For MNP blocking studies cells were seeded onto a 24 well plate and left to adhere for 24h. Cells were labelled with MNP as stated previously. For Frizzled blocked groups, cells were pre-incubated with 0.25ng/mL of anti-Frizzled 2 antibody (Santa Cruz) targeted to the extracellular Frizzled domain before labelling with Fz-MNP. Cells were washed with PBS, fixed with 90% Methanol for 10 mins then blocked with 2% BSA in PBS for 1h at room temperature. Cells were then stained with an anti-dextran antibody (Stem Cell Technologies) diluted 1:1000 in 0.1% BSA in PBS overnight at 4°C. Cells were then washed 3x with PBS and stained with anti-mouse FITC conjugated antibody (Sigma) diluted 1:1000 in 0.1% BSA in PBS for 1h at room temp. Cells were then washed 3X in PBS and counterstained with DAPI to visualise cell nuclei and Phalloidin-Atto 565 (Sigma) to visualise Actin filaments.

For β-catenin mobilisation experiments cells were seeded onto glass cover slips (SLS). At 24h post treatment media was aspirated, cells washed with PBS (sigma), then fixed with ice cold (90%) methanol (Fisher) for 10mins. Cells were permeabilised with 0.1% Triton-X in PBS (Sigma) for 10mins then then blocked in 2% BSA (Fisher) in PBS for 1h at room temp. Cells were then incubated with anti-active β-catenin antibody (Millipore) diluted 1:1000 in 0.1% BSA in PBS overnight at 4°C. Afterwards, cells were washed 3X with PBS, then incubated with anti-rabbit-FITC conjugated secondary antibody (Sigma) in 0.1% BSA in PBS for 1h at room temp. Cells were then washed 3X in PBS and counterstained with DAPI (Sigma).

Fluorescence microscopy was performed on a Nikon Eclipse Ti-S microscope with NIS elements software. All images are representative of 3 replicate samples. Pixel intensity analysis to assess nuclear β-catenin staining was performed using ImageJ v1.48s

### Luciferase reporter assays

Luciferase activity was assessed 48–72h post-transfection. Experiments were performed in 24-well plates with reduced serum (2.5%). For Wnt pathway blocking experiments, cells were cultured in reduced serum (2.5%) media containing either 0.1μg/mL Dkk1 or 50μM iCRT14 (R&D Systems). At each time point media samples were taken and analysed for secreted Gaussia luciferase activity using a luciferase flash assay kit (Thermo Pierce) on a Biotek Synergy 2 plate reader with automatic injection system controlled with Gen5 software. Luciferase activity was normalised to the total protein content of the cell lysates obtained using luciferase assay cell lysis buffer (Thermo Pierce) taken at the experimental end-point. Fold changes for each treatment were calculated relative to the cells only control.

### Gene expression analysis

At each time-point, media was aspirated and cells washed with PBS then lysed with TRI reagent (Sigma). Total RNA was extracted using the chloroform / isopropanol extraction method according to the manufacturer’s instructions. Reverse transcription was performed on 500ng total RNA (120ng for 1h samples) using a high capacity reverse transcription kit (Applied Biosystems). Quantitative PCR reaction mixes were prepared using 5μL of diluted sample mixed with a SYBR-Green master mix (Applied Biosystems) and commercial primers for COX2, c-Myc, NF-κB and GAPDH (Qiagen). Quantitative PCR was performed on a Stratagene MX3000P system with MxPro software. Gene expression was normalised to GAPDH and fold change gene expression was calculated using the ΔΔC_T_ method.

### Statistical analysis

All data is presented as means +/- SEM or SD. Statistical significance at 95% confidence level was determined using 1-way ANOVA with post-hoc Tukey tests using Mini-tab (v16) software.

## Results

### MNP Characterisation

The size and surface charge of anti-Fz MNP were characterised and compared to uncoated MNP. MNP size was shown to increase from approximately 299.7nm to 337.3nm after coating with antibodies. Also, MNP surface charge increased from approximately-18.9mV to-7.3mV after coating (Table 1).

**Table 1:** Particle size and surface charge after antibody coating.

Particle coating	Size (nm)	Zeta potential (mV)
Uncoated MNP	299.7±4.9	-18.9±1.6
Fz-MNP	337.3±3.8	-7.3±0.4

N = 3, error represents standard deviation.

### Fz-MNP specifically bind to Frizzled 2

**Fig 2:**
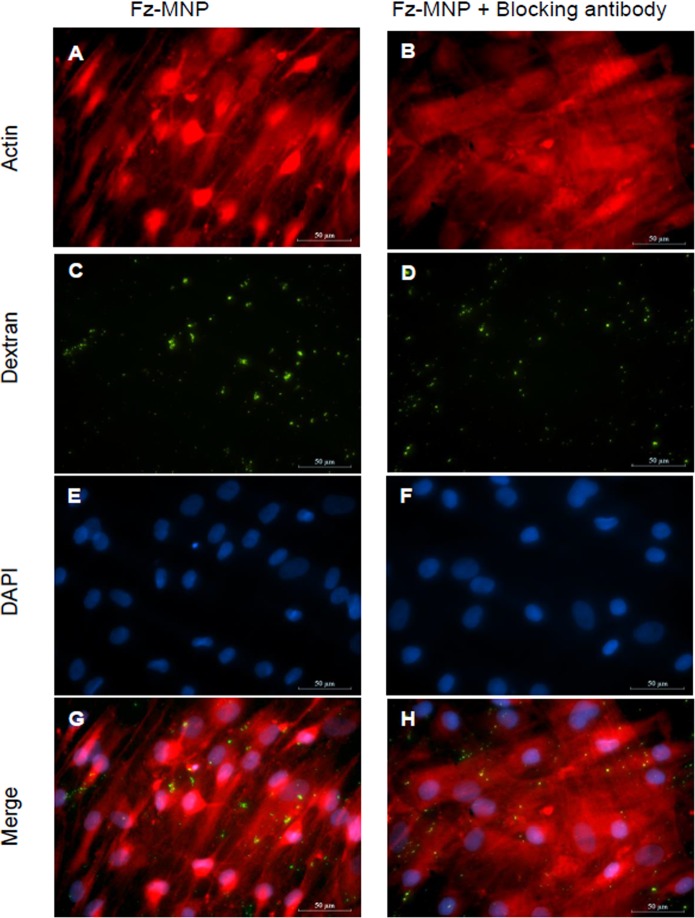
Frizzled antibody reduces Fz-MNP binding. Immunofluorescence images of cells labelled with Fz-MNP (A, C, E, G) or pre-blocked with anti-frizzled antibody before labelling with Fz-MNP (B, D, F, H). Cytoskeleton was visualised using Phalloidin (A, B), Anti-Dextran (C, D) was used to visualise Fz-MNP. Cell nuclei are shown by DAPI staining (E, F). Merged images are shown in G, H. Scale bar = 50μm. Images representative of n = 3.

To confirm the binding of anti-Fz MNP to hMSC and to assess the specificity of Fz-MNP for Frizzled receptors, an antibody blocking study was performed. Cells were labelled with Fz-MNP or pre-blocked with anti-Frizzled antibody to mask Fz-MNP binding sites before being labelled with Fz-MNP. Cell cytoskeleton was visualised using Phalloidin (Figs. 2A and 2B) and Fz-MNP were visualised using immunofluorescence by labelling the Dextran shell of the MNP with anti-Dextran antibodies (Figs. 2C and 2D). Cell nuclei were visualised using DAPI (Figs 2E and 2F). The overlay image of unblocked cells (Fig. 2G) shows that there is clear association of Fz-MNP with cells 1.5h after labelling. However the number of cell bound particles is reduced when cells are pre-incubated with anti-Fz antibody prior to labelling with Fz-MNP as shown by the overlay image of pre-blocked cells (Fig. 2H). Quantitation of bound particles using image analysis software indicated that Fz-particle binding was reduced by approximately 33% (data not shown).

### Fz-MNP signal through an LRP5/6 independent mechanism

**Fig 3:**
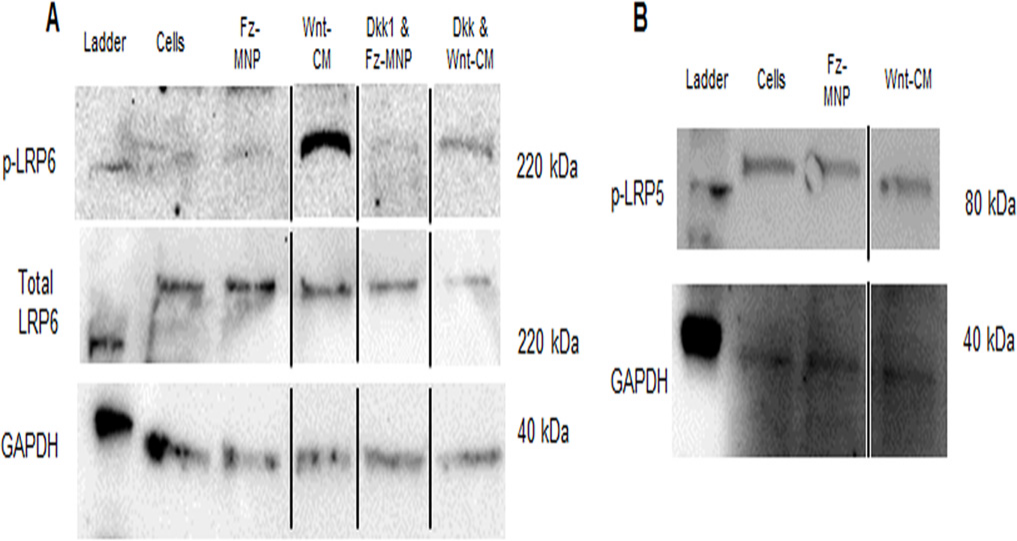
Fz-MNP signal through an LRP Independent mechanism. Treatment of hMSC with Frizzled-particles (Fz-MNP) did not result in phosphorylation of Wnt co-receptor LRP6. In contrast treatment with Wnt conditioned media (Wnt-CM) resulted in clear LRP6 phosphorylation which was blocked using the Wnt inhibitor Dickkopf related protein 1 (Dkk1). Non-treated cells displayed a basal level of Wnt co-receptor LRP5 phosphorylation (B). Treatment with Frizzled particles (Fz-MNP) or Wnt conditioned media (Wnt-CM) had no observable effect on the phosphorylation levels of LRP5. Black lines denote none adjacent lanes.

Western blotting was used to elucidate the mechanism of Wnt signalling activation by Fz-MNP by probing for the active (phosphorylated) forms of the Wnt co-receptor Low density lipoprotein (LDL) receptor-related protein 5 and 6 (LRP5/6). Fig. 3A shows that treatment of hMSC with Fz-MNP resulted in no changes in LRP6 phosphorylation whereas treatment with Wnt conditioned media resulted in clear phosphorylation of LRP6 after 3h which was predominately blocked with the addition of the LRP6 inhibitor Dickkopf 1 (Dkk1). LRP5 phosphorylation (Fig. 3B) was shown to be unchanged in response to either Fz-MNP or Wnt-CM. GAPDH was used as a loading control.

### β-catenin is mobilised in response to Fz-MNP

**Fig 4:**
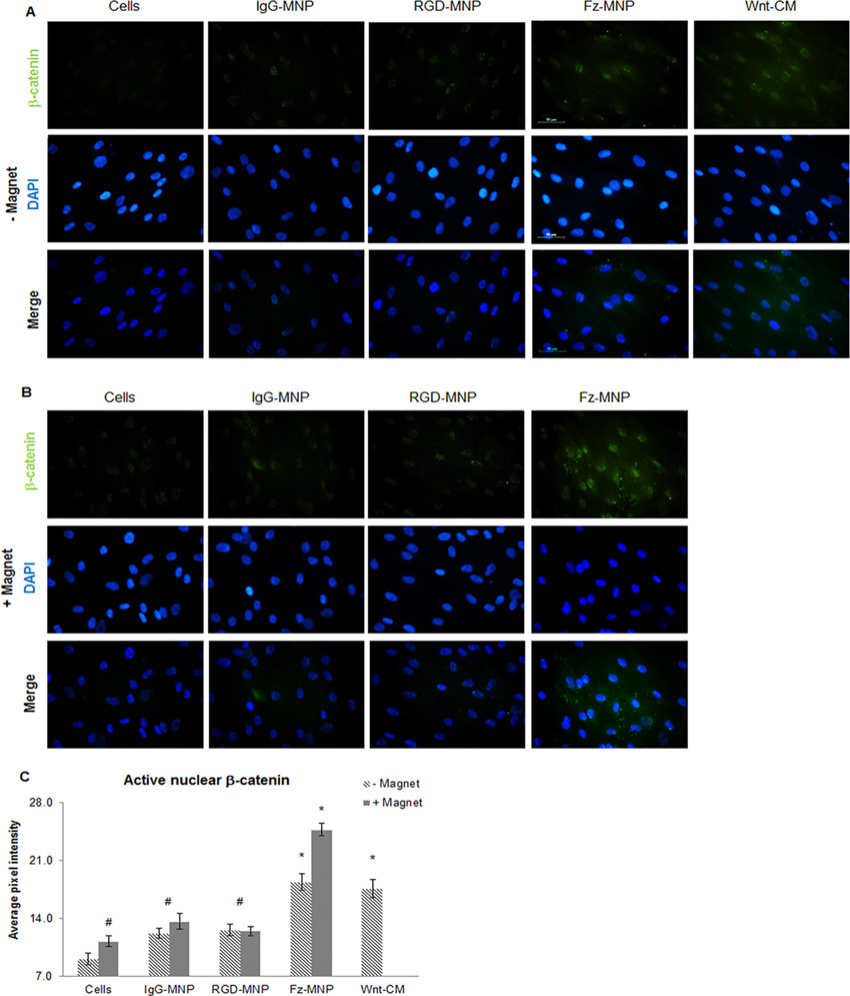
Frizzled-MNP promote β-catenin activation and mobilisation. Fluorescent images showing localisation of active β-catenin (Green) after 24h, DAPI was used to visualise cell nuclei (Blue). Non-treated cells (A) showed negligible β-catenin localisation. Cells treated IgG-MNP or RGD-MNP (A) resulted in a small non-significant increase in nuclear localisation, and addition of magnetic field (B) had no observable effect. Cells treated with magnetic field alone (B) showed a moderate increase in β-catenin localisation. Treatment with Anti-frizzled magnetic nanoparticles (Fz-MNP) without magnet (A) showed notable nuclear localisation with an added effect when used in conjunction with magnetic field (B). Treatment with Wnt-conditioned media (A) also showed notable nuclear β-catenin after 24h. Representative images, n = 3, scale bar = 50μm. Quantification of nuclear pixel intensity (C) indicated levels of nuclear (active) β-catenin. Treatment with magnetic field alone, IgG-MNP and RGD-MNP (with or without magnetic field) all caused similar increases in levels of nuclear β-catenin. Fz-MNP and Wnt-CM both increased β-catenin mobilisation to similar levels and an added effect was observed when Fz-MNP were used in conjunction with magnetic field. Average pixel intensities shown, n = 3, error bars represent SEM, * denotes p<0.05

To determine if Fz-MNP were causing Wnt signalling activation downstream of Frizzled, the mobilisation and nuclear localisation of the intracellular messenger β-catenin was investigated. β-catenin localisation was studied in hMSC after treatment with Fz-MNP with and without magnetic field or Wnt-CM. Fig. 4A shows that the non-treated cells displayed negligible β-catenin nuclear localisation after 24h. When cells were treated with anti-Fz MNP without magnetic field, clear nuclear localisation of β-catenin was observed and pixel intensity analysis of the cell nuclei confirmed a significant increase in nuclear localisation compared to non-treated control. Magnetic field stimulation of Fz-MNP labelled cells resulted in a further increase in β-catenin mobilisation (Fig. 4B). Pixel intensity analysis (Fig. 4C) also confirmed a significant increase in nuclear staining compared to cells + magnet control. Cells treated with Wnt-CM (Fig. 4A) showed noticeable nuclear localisation after 24h which was significant over non-treated control according to pixel intensity analysis. Control particles IgG-MNP and RGD-MNP (Fig. 4A) caused minor increases in β-catenin activation and no additive effect was observed with addition of magnetic field (Fig. 4B).

### Fz-MNP activate a TCF/LEF reporter

**Fig 5:**
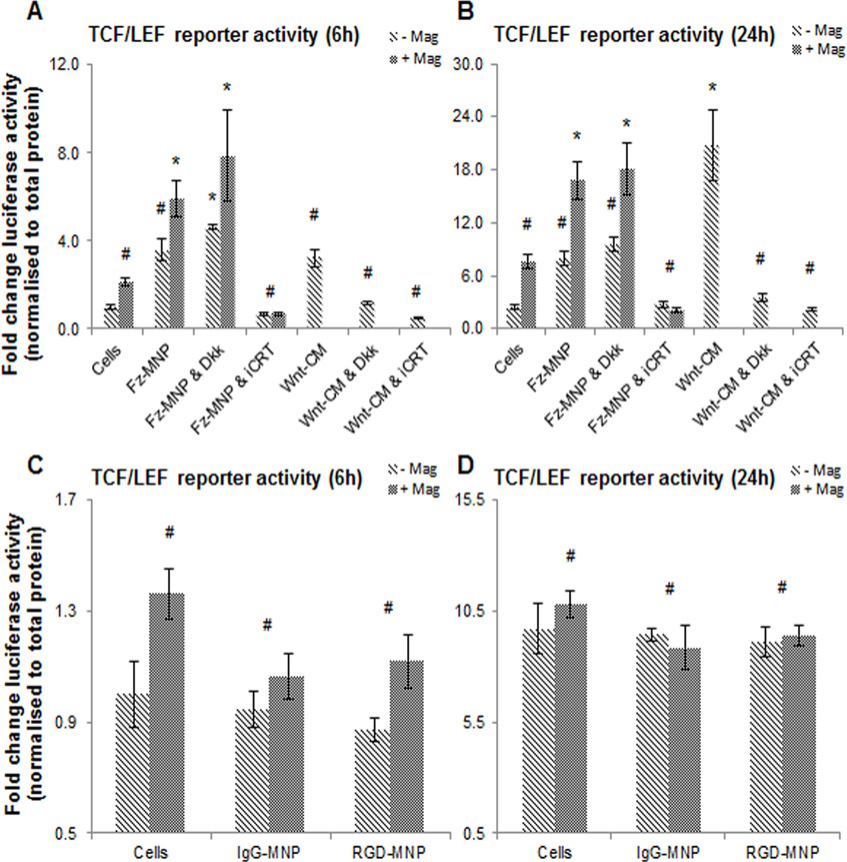
Anti-Frizzled particles activate a Wnt TCF/LEF luciferase reporter. TCF/LEF reporter activity from transiently transfected hMSC 6h and 24h after treatment. Magnetic field alone (hMSC + Mag) increased reporter activity at both time points (not significant) (A, B). Treatment with Frizzled particles (Fz-MNP) without magnetic field caused a noticeable increase in reporter activity over both time-points (trend only). An added significant increase in reporter activity was observed over both time-points when magnetic field was applied. Addition of the Wnt signalling blocker Dkk1 which inhibits LRP5/6 activation failed to prevent reporter activation by Fz-MNP. However activation was successfully blocked using the TCF/LEF inhibitor iCRT14, which inhibits Wnt signalling downstream of Frizzled. Treatment with Wnt-Conditioned Media increased reporter activity to a similar level as Fz-MNP (without magnet field) after 6h (A), with maximum activity observed after 24h (B). This effect was blocked using Dkk1 or iCRT14 with reporter activity being reduced to basal levels. Control particles coated with either Rabbit-IgG (IgG-MNP) or RGD peptide (RGD-MNP) caused no increase in reporter activity at 6h (C) or 24h (D) with or without magnetic field. Values represent mean fold change in luciferase activity relative to cells only with luciferase activity normalised to total protein. Error bars represent SEM, n = 4, * denotes p<0.05, # denotes p ≥ 0.05

A Gaussia luciferase reporter under control of a TCF/LEF promoter was used to directly asses the transcriptional activity of Wnt target genes in response to Fz-MNP, Wnt conditioned media and magnetic field stimulation over 24h. Transfected hMSC showed an increase (trend only) in luciferase reporter activity at 6h after treatment with Fz-MNP or Wnt-CM. An added significant increase in reporter activity was observed when cells were treated with Fz-MNP with magnetic field (Fig. 5A). The increase in reporter activity by Wnt-CM was successfully blocked using the Wnt LRP5/6 co-receptor blocker Dkk1 and iCRT14, a downstream Wnt signalling blocker that disrupts β-catenin association and interaction with TCF transcription factors which are required for expression of Wnt target genes. In contrast Dkk1 had no effect on reporter activity when cells were treated with Fz-MNP (with or without magnetic field) but these effects were blocked using iCRT14. At 24h (Fig. 5B) Fz-MNP treated groups showed a moderate increase in reporter activity (not significant) with an added significant effect when magnetic field stimulation was applied. Treatment with Wnt-CM also significantly activated reporter activity at 24h which was again blocked with Dkk1 or iCRT14. Treatment with Dkk1 again had no effect on reporter activity at 24h when used in conjunction with Fz-MNP whereas iCRT 14 also blocked reporter activation at 24h. Magnetic field treatment alone again increased reporter activity (not significant). (Figs. 5A and 5B). Control particles IgG-MNP and RGD-MNP were both found to have a negligible effect on reporter activation after 6h (Fig. 5C) and 24h (Fig. 5D).

### Fz-MNP alter stress response gene expression

**Fig 6:**
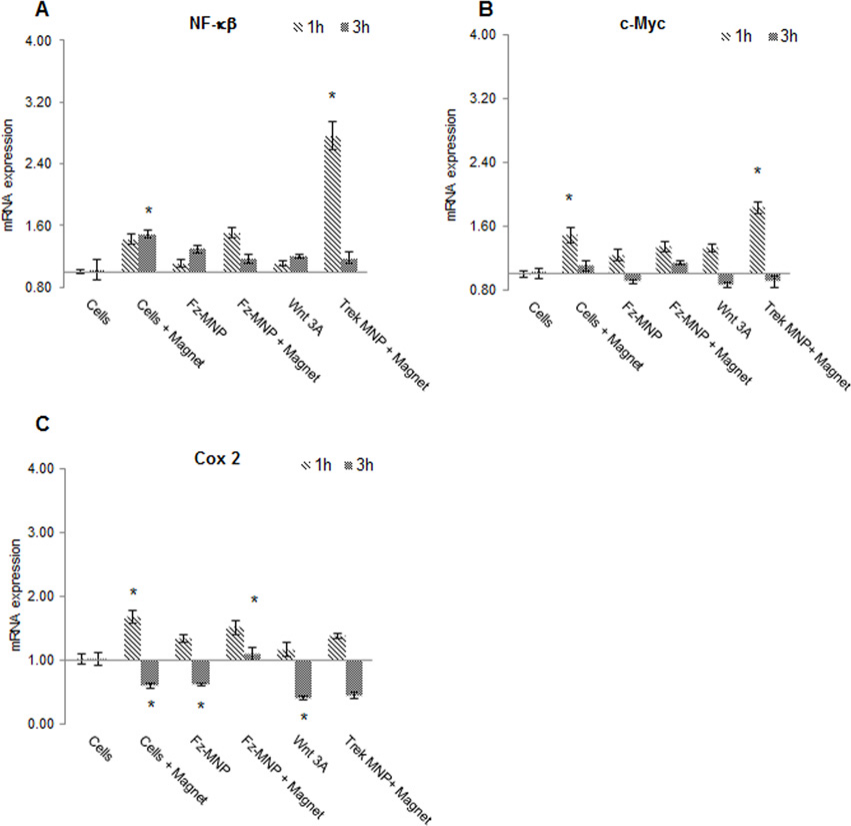
Anti-Frizzled MNP alter expression of Mechanosensitive genes. Gene expression analysis of mechano responsive genes (NF-κB, c-Myc, Cox 2) in response to anti-frizzled particles (Fz-MNP) or control particles (Trek-MNP) and magnetic field stimulation was evaluated using quantitative real-time PCR after 1h and 3h treatment. NF-κB gene expression (A) was increased by Trek-MNP (compared to cells + magnet control) after 1h. Magnetic field treatment alone also increased NF-κB expression (compared to cells only control) after 3h. Treatment with Fz-MNP or Wnt 3A both showed similar but minor shifts in NF-κB gene expression. c-Myc expression (B) was increased after 1h treatment with magnetic field treatment alone (cells + magnet) with an added increase when used in conjunction with Trek-MNP. Treatment with Fz-MNP or Wnt 3A again both showed similar but negligible shifts in c-Myc expression. COX 2 gene expression (C) was shown to be significantly increased after 1h by magnetic field treatment alone but a decrease in expression was observed after 3h. Treatment with Fz-MNP (without magnet) or Wnt 3A both caused significant decreases in COX 2 expression after 3h treatment. Treatment with Fz-MNP (+magnet) was shown to significantly increase COX 2 expression when compared to Cells + magnet control after 3h. Figures show mean fold change in gene expression normalised to GAPDH, Error bars represent SEM, n = 4, ANOVA p < 0.05 for all genes, * denotes p <0.05.

Gene expression analysis for stress response genes c-Myc, Cox2 and NF-κB was also performed at early time-points to investigate the levels of mechano-stimulation on cells. As predicted, treatment with the control particles (Trek-MNP) caused a significant up-regulation in NF-κB expression after 1h followed by a down-regulation in expression after 3h when compared to cells + magnet control groups (Fig. 6A). All other groups showed small but not significant levels of elevation at 1 and 3 hours compared to the TREK labelled response to magnetic field.

Investigations of responses in two other early response genes c-Myc and Cox2 showed low level variations in expression but none to the same extent as the NF-κB response observed to TREK coated particles with oscillating magnetic field. Small elevations in c-Myc expression were observed in all groups at 1 hour which fell by 3 hours with the most significant again observed in the TREK activated magnetic particle group (Trek-MNP). Similar levels of elevation were observed in Wnt3A activation as with particles alone, particles with magnetic field and magnetic field alone (Fig. 6B). A similar low level pattern of response was observed in expression of Cox2. Again small elevations in Cox2 were observed in all groups after 1 hour which fell after 3 hours with the greatest variation seen in magnetic field alone and magnetic field plus Fz-MNP groups. No significant difference was observed between experimental groups at one hour after treatment (Fig. 6C).

## Discussion

Wnt signalling is a crucial pathway controlling stem cell behaviour and in recent years has become an attractive target for modulation with potential applications in stem cell therapies. This study has demonstrated the feasibility of using anti-body functionalised magnetic nanoparticles to specifically target and stimulate Frizzled 2 receptors expressed by hMSC for the activation of Wnt/β-catenin signalling pathways.

The efficiency of MNP coating with antibodies was first assessed by examining changes in MNP characteristics. The antibody coating process resulted in an increase in particle size relative to uncoated particles; this could be attributed to the added protein layer around the coated MNP. Furthermore, the relative increase in MNP surface charge could also be attributed to the presence of protein on the particle surface. Previous work has also shown that particle coating with antibodies alters particle surface charge in a similar manner [33].

The binding specificity of Fz-MNP to Frizzled receptors was also assessed using a blocking study. Fz-MNP binding to cells was shown to be reduced when cells were pre-incubated with another Frizzled antibody. This infers that the Frizzled antibody successfully blocked the Fz-MNP binding sites on Frizzled, therefore reducing the available binding sites for Fz-MNP and consequently restricting particle binding to Frizzled receptors. This is an indicator that Fz-MNP are specific for Frizzled 2 receptors.

Our results show that treatment of hMSC with Fz-MNP caused no noticeable changes in LRP5/6 phosphorylation, indicating that Fz-MNP are not activating and signalling through LRP co-receptors. The canonical Wnt signalling co-receptor Low density lipoprotein (LDL) receptor-related protein 5/6 (LRP5/6) forms an active signalling complex with Frizzled and a Wnt ligand [34]. Upon receptor activation, LRP is successively phosphorylated at multiple sites by kinases such as GSK, a process which is mediated through Axin [11]. Phosphorylation of LRP in response to Wnt 3A has been shown previously [35]. In contrast to Fz-MNP, treatment with Wnt conditioned media (Wnt-CM) resulted in clear phosphorylation of LRP6 after 3h which was partially blocked with the addition of the LRP6 inhibitor Dickkopf 1 (Dkk1). Our results also show a basal level of LRP5 phosphorylation in untreated cells which is not altered by either Fz-MNP or Wnt-CM. This suggests that LRP5 is not the main co-receptor for transduction of Wnt signals and is in agreement with Perobner *et al* [36] who show that LRP6 not LRP5 is indispensable for canonical Wnt signalling. Altogether, our results suggest that Fz-MNP are activating Wnt signalling via a different mechanism to Wnt protein. One explanation for this observation could be receptor clustering and dimerization of Frizzled receptors caused by Fz-MNP which results in pathway activation. This mechanism has been proposed as an alternative route for Frizzled receptor activation by Carron *et al*. [37] who showed that dimerization of Frizzled receptors is enough to activate Wnt/β-catenin signalling. LRP independent signalling has also been shown to be involved with increased murine osteoblast proliferation in response to strain *in vitro* [38]. Further work could involve the investigation of the phosphorylation and activation status of LRP6 after targeting and stimulation with anti-LRP6 functionalised MNP to determine if this also leads to Wnt signalling activation. To determine if magnetic oscillation of Fz-MNP was causing Wnt signalling activation downstream of Frizzled, β-catenin mobilisation in response to MNP treatment was studied. We have demonstrated that both Fz-MNP and oscillating Fz-MNP are capable of downstream mobilisation of β-catenin into the nucleus. β-catenin mobilisation to the nucleus is a hallmark of active Wnt/β-catenin signalling and a precursor to transcription of Wnt responsive genes. This phenomenon is frequently used as a qualitative indicator of active Wnt signalling [39] [40]. Our results suggest that the binding action of Fz-MNP to Frizzled receptors is enough to initialise Wnt signalling and cause mobilisation of β-catenin. Furthermore, magnetic field stimulation of Fz-MNP labelled cells caused a further increase in β-catenin mobilisation and nuclear localisation. This suggests that there is an enhanced mechanoactivation of Frizzled receptors caused by movement of Fz-MNP in the magnetic field. Cells treated with Wnt-CM also showed noticeable nuclear localisation after 24h which was again significant over the non-treated control according to pixel intensity analysis of the nuclear β-catenin staining. These observations agree with the findings of Carthy *et al* who also showed that treatment with Wnt causes β-catenin mobilisation after 24h [41] [42]. Background mobilisation of β-catenin or background levels of fluorescence was also observed in control groups.

The critical downstream pathway of Wnt signalling has been shown to be transcriptional activation of TCF/LEF responsive genes [43] [44]. We have demonstrated that treatment with Wnt-CM results in an elevation of the TCF/LEF reporter constructs in transiently transfected hMSC. Furthermore we have gone on to demonstrate that mechanical activation of the Fz-MNP results in similar levels of activation of the downstream reporter. This observation confirms that Fz-MNP activate Wnt-responsive elements after a short time period and have similar effects as Wnt-CM on pathway activity. The increase in Wnt luciferase reporter activity by Wnt-CM was potently blocked using the Wnt LRP5/6 co-receptor blocker Dkk1; this can be expected as signalling activation by Wnt protein requires LRP co-activation. However, in our experiments Dkk1 had no effect on the blocking of reporter activity (at both time-points) when cells were activated through the oscillating Fz-MNP. This suggests that Fz-MNP are signalling through an LRP independent mechanism. This result is in agreement with the Western blotting data which showed no noticeable increase in LRP5/6 phosphorylation after treatment with Fz-MNP which would indicate receptor activation. This again suggests an alternative mode of Wnt/β-catenin activation by Fz-MNP which warrants further investigation. At 24h Fz-MNP treated groups remained elevated. This is further evidence of a level of mechanoactivation of Wnt signalling and demonstrates that Fz-MNP are capable of causing sustained Wnt pathway activation. Treatment with Wnt-CM also significantly activated reporter activity to comparable levels as Fz-MNP (with magnet). Taken together, this would suggest that a lag phase or threshold exists after stimulation with Wnt-CM that must be overcome before Wnt signalling activity peaks. This observation is in agreement with work from Carthy *et al* who showed activation of a Wnt TCF reporter after 24h treatment with recombinant Wnt 3A [41]. Although Dkk1 was unable to block Wnt pathway activation by Fz-MNP, reporter activation by both Fz-MNP and Wnt-CM was successfully blocked using a downstream Wnt signalling blocker- iCRT-14, which acts by disrupting β-catenin’s association and interaction with TCF transcription factors which is required for the expression of Wnt target genes. Control particles IgG-MNP and RGD-MNP were used to assess the effects of generic membrane stimulation on pathway activation. In our experiments both control particles were found to have no activating effects on reporter activity over both time-points studied.

Finally, gene expression analysis was performed over early-time-points to investigate generic mechano-stimulation of stress response genes which have previously been shown to respond to mechanical stimulation. C-Myc is an oncogene associated with stress response; it has also been identified as a Wnt responsive gene and expression of c-Myc has been shown to increase in response to Wnt treatment [45]. Low levels of up-regulation in all groups are observed after one hour e.g. Fz-MNP treatment (with or without magnetic field) and Wnt 3A treatment all up-regulate c-Myc expression after 1h to similar levels. This is comparable to results from Gujral *et al* [46] who showed c-Myc expression increases in the first 3h after Wnt stimulation in HEK293 cells. Furthermore, in this experiment the expression profile of Fz-MNP matches the expression profile produced by Wnt 3a closely, whereas treatment with control particles targeted to the Trek-1 ion channel (Trek-MNP) produced a broadly different expression profile to Wnt3A and Fz-MNP with Magnet (at 1h time-points). This is an indication that the Fz-MNP and Wnt 3A are having a similar effect on c-Myc gene expression.

COX2 expression has been shown to increase in response to cytotoxic stress e.g. cytokines, endotoxins, γ-radiation [47] [48]. Recent evidence has also shown that the COX-2 promoter harbours TCF/LEF response elements and that activation of canonical Wnt signalling by lithium or Wnt 3A results in increased COX2 mRNA expression [49]. Our results again show low levels of expression elevation at 1 hour in all experimental groups without a clear pattern emerging.

In contrast, our investigation of the levels of NF-κB show a clear elevation in response to our control group labelled with TREK particles. NF-κB is a stress response gene activated when cells are subjected to physiological stresses e.g. mechanical stimulation [50] [51]. The expression profile of NF-κB is similar when cells are treated with either Wnt 3A or Fz-MNP without magnet with these treatments causing slight increases in NF-κB expression (trend only). In contrast, treatment with control particles (Trek-MNP) caused a significant up-regulation in NF-κB expression after 1 hour when compared to cells plus magnet control group. This again demonstrates the differing outcomes when cells are treated with either Fz-MNP or Trek-MNP.

## Conclusions

Wnt signalling pathways are important for the regulation of cell behaviour and there is increasing interest in the development of modulators of Wnt signalling. These tools may have therapeutic potential in fields such as stem cell science, mammalian development, cell and tissue engineering and cancer. One way of controlling cell signalling is by using bio-functionalised MNP. To date no one has attempted to locally target and stimulate Wnt receptors in order to modulate Wnt signalling. Our work has shown that it is possible to tag Frizzled receptors on hMSC with antibody-functionalised nano-particles and use an oscillating magnetic field to impart focused mechanical stimulation of Frizzled receptors. This strategy has enabled the unconventional activation of Wnt signalling pathways in hMSC. Immunocytochemistry has demonstrated the targeting specificity of Fz-MNP with the use of blocking antibodies. Western blotting has indicated that Frizzled receptor and subsequent Wnt signalling activation with Fz-MNP is independent of LRP. Immunofluorescence studies showed mobilisation of the Wnt signalling messenger β-catenin in response to Fz-MNP treatment and activation of a Wnt signalling reporter construct. Gene expression analysis has shown differential expression of stress response genes in response to Fz-MNP and Wnt treatment. Taken together, these results suggest that Fz-MNP and Wnts are acting via different mechanisms to activate Wnt/β-catenin signalling. The mechanism behind signal activation through Fz-MNP and the effects on hMSC fate and differentiation requires further investigation. Also the remote activation of other Frizzled receptors and co-receptors for the modulation of Wnt pathways using MNP technology should be investigated. The development of this technology raises the possibility of remotely controlling Wnt signalling and consequently the control of stem cell behaviour.

## Supporting Information

S1 File Supporting information File S1. Graph and table data.
(XLSX)

S2 File Supporting information File S2. Western blots.
(DOCX)
