# Supplementary material for: Remote Activation of the Wnt/β-Catenin Signalling Pathway Using Functionalised Magnetic Particles
Source: PLoS One. 2015 Mar 17;10(3):e0121761. doi: 10.1371/journal.pone.0121761 (PMC4363733; doi:10.1371/journal.pone.0121761)
Supplement: S2 File — Western blots. (DOCX) [file pone.0121761.s002.docx]

**
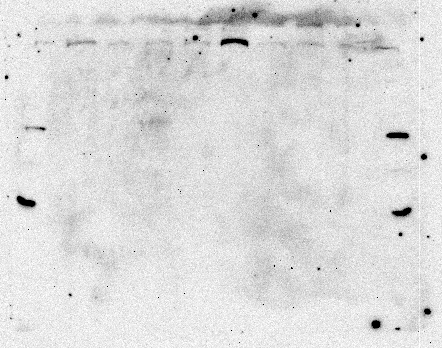
Figure 3A – Whole blots**

Figure 3A: p-LRP6


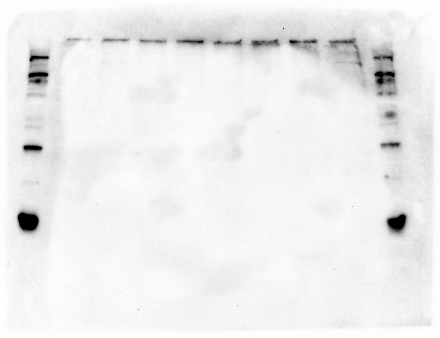


Figure 3A: Total LRP6


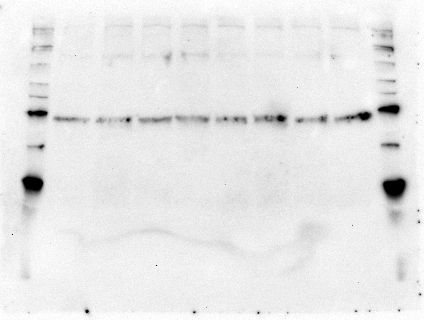


Figure 3A: GAPDH

**
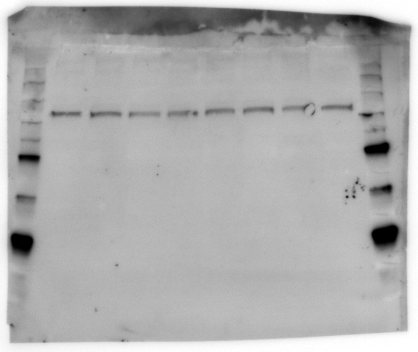
Figure 3B – Whole blots**


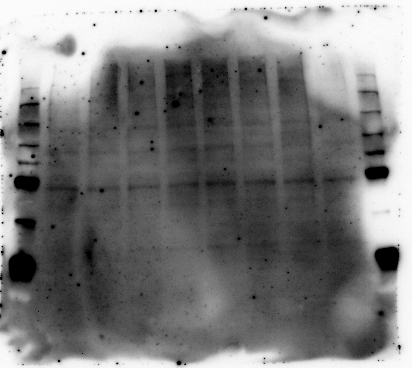


Figure 3B: GAPDH

Figure 3B: p-LRP5
